# Invasion of superbugs: Cockroach-driven outbreak of multidrug-resistant *Enterobacter* in an ICU

**DOI:** 10.1017/ash.2024.425

**Published:** 2024-10-01

**Authors:** Jennifer Hanrahan, Nicholas Schouten, Steven H. Fyffe, Annette Jencson, Stephanie Stroever

**Affiliations:** 1 MetroHealth Medical Center, Cleveland, OH, USA; 2 Macon and Joan Brock Virginia Health Sciences, EVMS Medical School at Old Dominion University, Norfolk, VA, USA; 3 Texas Tech University Health Sciences, Lubbock, TX, USA

## Abstract

**Objective:**

To describe factors in an outbreak of multidrug-resistant *Enterobacter cloacae* (MRE) in an intensive care unit (ICU) over a 20-month period including the likely contribution of cockroaches to the outbreak.

**Design:**

This is a retrospective descriptive study.

**Setting:**

ICU in an urban hospital.

**Patients:**

All patients admitted to the ICU during the 20-month outbreak period were included in the study.

**Interventions:**

Infection prevention interventions included contact isolation, hand hygiene, dedicated patient equipment, environmental cultures, and pest control.

**Results:**

25 patients were identified as being colonized or infected with MRE. Relatedness of the outbreak strain and strains found in cockroaches was demonstrated by pulse field gel electrophoresis. Standard IP interventions did not have an impact on the outbreak until pest control was added. Once additional pest control measures were put in place, the outbreak ended.

**Conclusions:**

Insects have a potential role in transmission of pathogens in hospitals and their role should be considered when outbreaks are being investigated.

## Introduction

*Enterobacter* species are common pathogens causing healthcare-acquired infections (HAIs). Most strains have innate antibiotic resistance and easily acquire resistance determinants.^1,2^ Outbreaks with *Enterobacter* species can go on for years secondary to environmental contamination and cross-transmission between patients.^3–5^ Infection control measures such as hand hygiene (HH), contact isolation (CI) dedicated patient equipment, and selective antibiotics are often implemented to halt outbreaks.^3–5^ As in other nosocomial infections, understaffing and overcrowding can contribute to cross-transmission.^6^ Furthermore, contamination of multi-dose vials,^7^ feeding solutions or formula,^13^ and hospital equipment have been described.

Hospitals outside the U.S.^9–14^ have identified cockroaches as potential vectors of transmission in healthcare settings and highlighted *Enterobacter* species as frequently isolated pathogens from cockroaches found in hospitals along with other pathogens.^11–14^ However, there have been few publications discussing the role of insects in environmental contamination in the hospital. The objective of this study is to describe the likely contribution of cockroaches to a healthcare-associated outbreak of multidrug-resistant *Enterobacter cloacae* (MRE).

## Methods

### Study design

This study is a descriptive study of an outbreak that occurred in an ICU. Approval was obtained from the medical center’s institutional review board prior to initiating the study.

#### Study population

All individuals admitted to the ICU during the study period were included. Three of 131 patients were excluded because they were immediately transferred, and culture data were unavailable.

#### Outbreak

A possible outbreak was noted when infection control practitioners noted two individuals in adjacent rooms with MRE, and cross-transmission was assumed. HH, CI, and dedicated patient equipment measures were instituted. Once the outbreak was identified, all patients were placed in CI on admission. Screening cultures were performed from rectal swabs and clinical cultures, and patients were removed from CI if no indication was found once results were available. Despite this, two additional cases were noted raising concerns for a point-source outbreak.

An environmental investigation was carried out that included cultures from patient rooms, water, a mobile X-ray machine, and ventilator equipment. Room surfaces that yielded MRE included: bedside cabinets, mattresses, bed frames, trashcan lids, ventilators, and suction controls. Multi-dose vials and protein supplements were cultured and were negative. Artificial fingernails and jewelry were prohibited and cultures of healthcare-workers hands were negative for MRE.

Cleaning procedures were reinforced and rooms were terminally cleaned with quartenary ammonium and kept unoccupied until negative cultures were obtained. Some rooms had to be cleaned repeatedly. Because of difficulty in eradicating the organism after repeated cleaning, resistance to cleaning solutions was suspected, but both quaternary ammonium and bleach solutions inhibited growth of MRE according to use-dilution tests performed in the microbiology laboratory. Because resistance to cleaning solutions was not demonstrated, quartenary ammonium use continued.

A few rooms housing critically ill patients were noted to have negative cultures, but when they remained empty and were recultured, were positive again for MRE. During follow-up discussions with nurses to identify possible sources, one observed that there had been an occasional cockroach in patient rooms. These were rare, and not perceived to be a serious problem. A cockroach was caught, placed on sheep blood agar, and walked on the plate for several hours. MRE was isolated along with several other resistant nosocomial pathogens (MDR pseudomonas, MRSA, MSSA, *Aspergillus*, and coagulase-negative staphylococcus.)

Pest control specialists reinforced measures used against insects and no new patients were found to be colonized with MRE for three months. No nests were identified, but the pest control specialist thought that roaches were most likely coming from drains and concentrated on these for pest control in addition to standard procedures.

**Figure 1.**
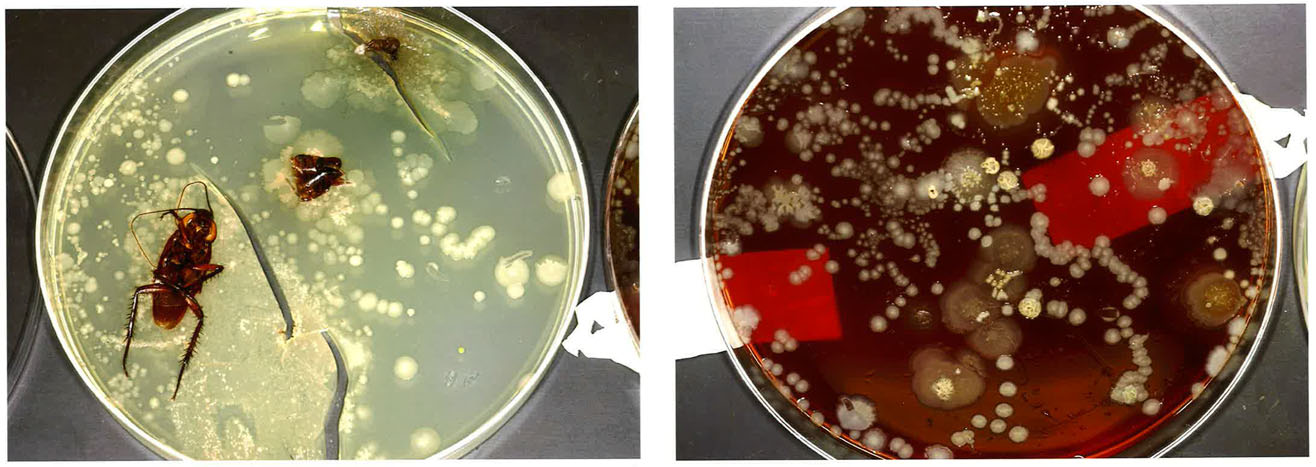
Cockroach with Organisms Cultured from the Roach.

Thirteen months after initial cases were identified, another roach was found in the ICU. It was cultured and yielded the same organism as demonstrated by pulse field gel electrophoresis (PFGE). One week later, a new patient was identified as colonized with MRE. Pest control was again reinforced, and the last patient case was noted shortly thereafter. No further patients were identified as colonized or infected following this last patient (Figure 1).

### Laboratory methods

Environmental samples obtained by ICPs using sterile swabs or gauze soaked with sterile water were labeled and placed in sterile containers for culture. Thioglycolate broth was added, and specimens were vortexed and incubated 18–24 hours at 37°C. The liquid was vortexed again, swabbed onto 5% sheep blood agar and MacConkey agar, and incubated again to be examined daily for 5 days. MRE was resistant to cephalosporins, piperacillin-tazobactam, trimethoprim-sulfamethoxazole, ciprofloxacin, and aminoglycosides.

## Results

On chart review, a patient was found who had been transferred from another hospital 5 months before 2 initial cases were identified. During the outbreak, 131 patients were admitted to the ICU, 3 of whom were present less than 24 hours and not cultured. Of the remaining 128 patients, 25 were identified as either infected or colonized with MRE. This constituted 19.5% of 128 admissions during the outbreak period. The mean time to colonization or infection with MRE was 14.5 ± 10.7 days with a minimum time of 3 days. Of the clinical isolates, 2 patients had positive stool cultures, 18 had wound cultures, 4 had blood cultures and 1 had a urine culture. Standard culture methods were used to identify MRE. The turnaround time from the microbiology laboratory was 3–5 days to obtain identification and sensitivity. Length of stay (LOS) for patients identified as cases as 30.20 ± 25.99 days (D) and 18.37 ± 8.28 D for controls. There was no statistical difference in LOS.

Isolates from roaches and patient specimens were sent to a reference laboratory that performed PFGE. Testing confirmed that the cockroach isolate, environmental specimens, and clinical isolates from patients all had identical patterns on PFGE, while strains unrelated to the outbreak showed different patterns.

## Discussion

In this outbreak, usual infection prevention (IP) methods including HH, careful cleaning of equipment, designated single use items, and CI were reinforced. Multiple surfaces yielded positive cultures after cleaning. Ultimately, conventional interventions were not effective in ending the outbreak.

Cockroaches were found carrying MRE and multiple environmental surfaces were contaminated. To determine strain relatedness, samples were sent to a reference lab for PFGE. Results took longer than they would now. A current outbreak would be investigated with whole genome sequencing which could identify strain relatedness earlier, but would not have identified the source of the problem.

Cockroaches have rarely been reported in hospitals outside the U.S. as vectors of transmission but our outbreak is not likely a unique situation. The role of insect vectors may be underestimated. Cockroaches are notoriously difficult to control, and after initial pest control measures were reinforced, there was an immediate decrease in cases for 3 months. At this point, an additional patient was found positive for MRE, and additional pest control measures were implemented. After 20 months, no additional cases were found. It is suspected that the cockroaches either carried MRE and mechanically transferred the organism to surfaces, or may have defecated on surfaces and contaminated them in this manner. Lapses in insect control have not been described as associated with HAIs secondary to cockroaches in the US, but they are likely contributing to environmental contamination and cross-transmission in healthcare settings.^13^ It is essential that infection control protocols include vigilance and eradication efforts to prevent perpetuation of outbreaks secondary to hospital insects. Additionally, investigation of outbreaks should consider insects as vectors if identified in patient care areas. The solution to this outbreak came from discussion with frontline staff when an astute nurse identified cockroaches as a potential problem. Any infection control problems should be discussed with frontline staff.
